# Early-Stage Development of an Anti-Evaporative Liposomal Formulation for the Potential Treatment of Dry Eyes

**DOI:** 10.1021/acsptsci.3c00147

**Published:** 2023-09-21

**Authors:** Janika Jäntti, Tuomo Viitaja, Julia Sevón, Tatu Lajunen, Jan-Erik Raitanen, Cordula Schlegel, Mira Viljanen, Riku O. Paananen, Jukka Moilanen, Marika Ruponen, Filip. S. Ekholm

**Affiliations:** † School of Pharmacy, University of Eastern Finland, P.O. Box 1627, FI-70211 Kuopio, Finland; ‡ Department of Chemistry, University of Helsinki, P.O. Box 55, FI-00014 Helsinki, Finland; § Ophthalmology, University of Helsinki and Helsinki University Hospital, Haartmaninkatu 8, FI-00290 Helsinki, Finland; ∥ Faculty of Pharmacy, University of Helsinki, FI-00790 Helsinki, Finland

**Keywords:** anti-evaporative, biophysical characterization, dry eye disease, liposomal formulation, tear film lipids, preclinical screening

## Abstract

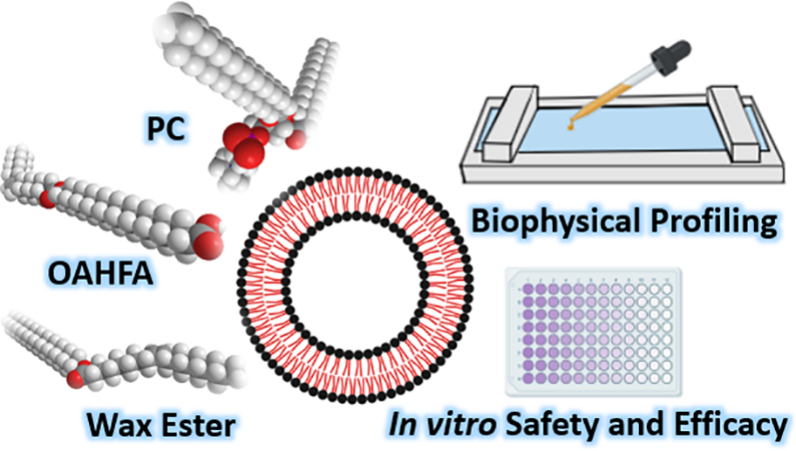

Dry eye disease (DED), the most common ocular disorder, reduces the quality of life for hundreds of millions of people annually. In healthy eyes, the tear film lipid layer (TFLL) stabilizes the tear film and moderates the evaporation rate of tear fluid. In >80% of DED cases, these central features are compromised leading to tear film instability and excessive evaporation of tear fluid. Herein we assess the potential of liposomal formulations featuring phosphatidylcholines and tailored lipid species from the wax ester and *O*-acyl-ω-hydroxy fatty acid categories in targeting this defect. The developed lead formulation displays good evaporation-resistant properties and respreadability over compression–expansion cycles in our Langmuir model system and a promising safety and efficacy profile *in vitro*. Preclinical *in vivo* studies will in the future be required to further assess and validate the potential of this concept in the treatment of DED.

## 1 Introduction

Dry eye disease (DED) affects millions of people on a global scale.^1^ While already prevalent, the incidence is expected to rise significantly in the future as the modern societal trends are closely connected to the risk factors of the disease (e.g., multiscreen lifestyle, aging population etc.).^1−8^ The current mainstream DED-treatments consist of artificial tears and ocular lubricants (both lipid based and others). In severe DED cases, anti-inflammatory agents are further utilized to treat the resulting ocular inflammation. While aqueous ocular lubricants are the most widely used treatments, they are unable to successfully target the tear film instability and excessive tear evaporation defect (present in >80% of DED-cases).^9−14^ Thus, they offer only a brief alleviation of symptoms even upon frequent application. Anti-inflammatory agents, on the other hand, are associated with several adverse effects (e.g., ocular hypertension and cataracts) in long-term use, which poses a challenge when the chronic nature of some instances of DED is considered.^15−17^ Therefore, the expert ophthalmologists have called for the immediate development of improved DED treatments capable of successfully targeting the tear film instability and excessive tear evaporation defects associated with the disease.^18^ Within the scientific community, there is a growing consensus that strategies focusing on the tear film lipid layer (TFLL) hold significant potential for improving the treatment outcomes. This is because the TFLL in healthy eyes is responsible for the upkeep of tear film stability and an optimal tear evaporation rate,^19^ features that are compromised in dry eye patients because of structural/functional defects.

The TFLL contains >200 lipid species from distinct lipid classes, which through their collaborative action sustain and regulate its structure and function.^20−23^ These factors need to be taken into consideration when developing a treatment concept focused on the replenishment of TFLL action. In order to minimize potential disruptive effects, lipid species with biophysical profiles matching those found in an intact and fully functioning TFLL would be ideal. Recent work has showcased that out of the many unique lipid classes found in the TFLL, the wax esters (WEs) and *O*-acyl-ω-hydroxy fatty acids (OAHFAs) hold significant potential from the DED treatment perspective. In more detail, they display the characteristic biophysical profiles required to promote active spreading of lipids at the aqueous interface (tear film stabilizing action) and enhance the evaporation resistance of the formed film (moderation of evaporation rate).^24,25^ In our most recent work, we identified a promising lipid composition comprising the WE behenyl oleate (BO) and OAHFA 20-oleoyloxy-eicosanoic acid (20:0/18:1-OAHFA, later referred to as 20-OAHFA) which spread efficiently at the aqueous interface to yield a lipid film with excellent evaporation-resistant properties at physiological ocular surface pressure (20–40 mN/m) and temperature (35 °C).^26^ In more detail, the 20-OAHFA was tailored to maintain the functional characteristics of the average TFLL OAHFA ((21*Z*)-29-oleoyloxynonacos-21-enoic acid used as a model, i.e., 29:1/18:1-OAHFA, later referred to as 29-OAHFA), that is, to capture its surface-active properties, phase transition behavior, and evaporation-resistant function in an economically more sensible form.^24,25^ This would be more appealing from an industrial upscaling perspective as the length of the synthetic routes are considerably shorter than those required to reach the naturally occurring OAHFAs (2–3 steps for 20-OAHFA^24^ vs 9–10 steps for 29-OAHFA).^25^ BO, on the other hand, is a commercially affordable endogenous TFLL lipid that when applied together with the 20-OAHFA species significantly enhances the evaporation resistance of the formed film. We therefore envisioned that incorporation of these species in a treatment for DED would allow replenishment of proper TFLL structure and restoration of its key functions with minimal disruptive effects on the dynamic behavior of this biological membrane. This would altogether translate into an efficient treatment strategy that values patient safety. However, due to the highly lipophilic nature of BO and 20-OAHFA (low solubility in aqueous solutions), the development of a formulation allowing their topical administration to the surface of the eye posed a challenge. Herein, we focused on overcoming this challenge through an early stage development and screening campaign centered on liposomal formulations incorporating 20-OAHFA and BO. On a general level, liposomal formulations have emerged as a viable alternative for various ophthalmic applications ranging from prolonging the time of drug action to enhancing the drug delivery to specified compartments of the eye, and more importantly, have already found a home in dry eye products to a certain extent.^27−32^ Nevertheless, the development of a liposomal formulation sustaining the functional features of BO and 20-OAHFA was not straightforward. Through the generation of a dedicated *in vitro* screening platform for assessment of key properties and a substantial screening campaign, we arrived at promising liposomal formulations which sustain the efficient spreading and anti-evaporative features of the BO/20-OAHFA mixtures, promote the recovery of damaged ocular surface cells, and can be tailored to meet the pharmaceutical requirements for ophthalmic products in terms of flow properties, particle size, pH, osmolality, and safety.

## 2 Results

### 2.1 Development and Characterization of Liposomal Formulations

Initial attempts at developing liposomal formulations from BO and 20-OAHFA alone proved unsuccessful, and therefore a third component was needed. The choice fell on phospholipids for two reasons: (1) Phospholipids are known to form liposomes in aqueous solutions which can integrate hydrophobic compounds into the phospholipid bilayer. (2) Phospholipids constitute up to 12% of the total lipid content of human tears which make them an excellent overall choice for this application.^20^ In order to further refine our selection of phospholipids, we considered that cationic species might interact too strongly with the anionic cell surfaces, and thus, we focused on the use of neutral zwitterionic phosphatidylcholines (PCs). It is worth noting that PCs of various chain lengths and saturation degrees have been reported in human Meibum thus suggesting that they are well-tolerated and therefore expected to have a minimal intrusive or harmful effect on the structure and function of the tear film lipid layer.^33^ From the formulation development perspective, it is widely recognized that the carbon chain length of PLs affects the formulation stability and incorporation efficiency/capability of lipophilic species. Therefore, we decided to assess a series of PCs with variations in the chain length. The PCs included were 1,2-ditetradecanoyl-*sn*-glycero-3-phosphocholine (14:0-PC; later termed DMPC), 1,2-distearoyl-*sn*-glycero-3-phosphocholine (18:0-PC; later termed DSPC), and 1,2-diarachidoyl-*sn*-glycero-3-phosphocholine (20:0-PC; later referred to as DAPC). In addition to the factors mentioned above, the 14:0–20:0-PC range was chosen in order to explore formulations in which the phase transition temperature of the PC is below (DMPC) or above (DSPC and DAPC) that of the ocular surface. In other words, DMPC would enable the development of thermosensitive formulations featuring a liquid and disordered phospholipid bilayer phase,^27^ whereas DSPC and DAPC would enable the development of formulations with an ordered gel-like phase. We envisioned that screening both types of PCs would enable identification of the boundaries within which the successful formulation of 20-OAHFA and BO is possible and a good base for assessing the effects of PCs on the functional properties of these species.

A large number of liposomal formulations were prepared through the thin film hydration method by varying the amounts of PCs, 20-OAHFA and BO. Table 1 contains a summary of the formulation screening campaign. While the thin film hydration method is generally considered a straightforward protocol for generation of liposomes, incorporation of lipophilic components at relatively high overall concentrations resulted in challenges during the formulation process. In more detail, challenges were encountered during the hydration of the lipid film, at the ultrasonication step, and in some cases, the final appearance and properties of the formulation did not meet our selection criteria (e.g., viscous solutions with a more gel-like appearance, not suitable for topical administration as an eye drop due to poor flow properties). The formulations in which these challenges were noted are marked with an “A” in Table 1 and were not considered eligible.

**Table 1:** Eligibility Criteria and Results from the Formulation Development Campaign

lipid composition	mass ratios	molar ratios	% (W/V)	eligibility (+/−)	observations^a^
DMPC/BO/20-OAHFA	40:3:1	35:3:1	≥20:1.5:0.5	–	A
			≤13:1:0.33	+	
	30:3:1	26.2:3:1	≤15:1.5:0.5	+	
	7:1:0	6.1:1:0, 6.1:0:1	≤12:1.75:0, ≤ 12:0:1.75	+	B
	7:0:1				
	6:1:0, 6:0:1	5.2:1:0, 5.2:0:1	≤12:2:0, ≤ 12:0:2	+	B, C
	20:3:1	17.5:3:1	≤10:1.5:0.5	+	B
	5:1:0, 5:0:1	4.4:1:0, 4.4:0:1	≤10:2:0, ≤10:0:2	+	B
	16:3:1	14:3:1	≤12:2.25:0.75	+	B
	8:1:1	7:1:1	≤12:1.5:1.5	+	B, C
	4:0:1, 4:1:0	3.5:0:1, 3.5:1:0	≤12:3:0, ≤12:0:3	+	B, C
	12:3:1	10.5:3:1	≤12:3:1	+	B, C
	3:0:1, 3:1:0	2.5:1:0, 2.6:0:1	≤12:4:0, ≤12:0:4		B, C
					
DSPC/BO/20-OAHFA	6:1:1	4.5:1:1	≥12:2:2	–	A
			≤6:1:1	+	
	4:1:1	3:1:1	≤2:0.5:0.5	+	
	2:1:1	1.5:1:1	≤1:0.5:0.5	–	A, D
					
DAPC/BO/20-OAHFA	12:1:1	8.4:1:1	≤3:0.25:0.25	+	
	10:1:1	7.0:1:1	≥5:0.5:0.5	–	A
	8:1:1	5.6:1:1	≥4:0.5:0.5	–	A
	7:1:1	4.9:1:1	≤3.5:0.5:0.5	+	
	6:1:1	4.5:1:1	≥3:0.5:0.5	–	A
			≤1.5:0.25:0.25	+	
	4:1:1	2.8:1:1	≤2:0.5:0.5	+	
	2:1:1	1.4:1:1	≤1:0.5:0.5	–	A, C, D

a A: Concentration-dependent challenges noted during formulation step. B: The use of either BO, 20-OAHFA or a combination of the two did not result in changes in the phase transition behavior. C: alterations in the phase transition behavior was noted compared to the listing above. D: Additional endothermic peak detected at the temperature area close to the melting point of BO.

A general trend discovered was that pursuing liposomal formulations with higher overall lipid concentrations was accompanied by an increased likelihood of encountering difficulties during the formulation process. Universal total lipid concentration limits could not be identified, as the properties displayed by the formulations were dependent on the utilized PC. PCs with longer chain lengths (DSPC, DAPC) were found to tolerate total lipid concentrations lower than those of PCs with shorter chain lengths (DMPC). With DMPC based formulations, the formulation process was straightforward and eligible formulations with total lipid concentrations up to 17% could be successfully developed. With DSPC and DAPC based formulations, the hydration step was found to be more time-consuming than with DMPC formulations and a tendency toward formulations with gel-like appearance was initially noted. Nevertheless, by optimizing the formulation composition (lowering the total lipid concentration) and preparation process (performing sonication above the gel-to-liquid phase transition temperature (*T*_c_)), these challenges could be overcome. At the end, eligible DSPC based formulations with total lipid concentrations up to 8% could be successfully developed while DAPC allowed the development of eligible formulations with total lipid concentrations up to 3–4%.

**Figure 1:**
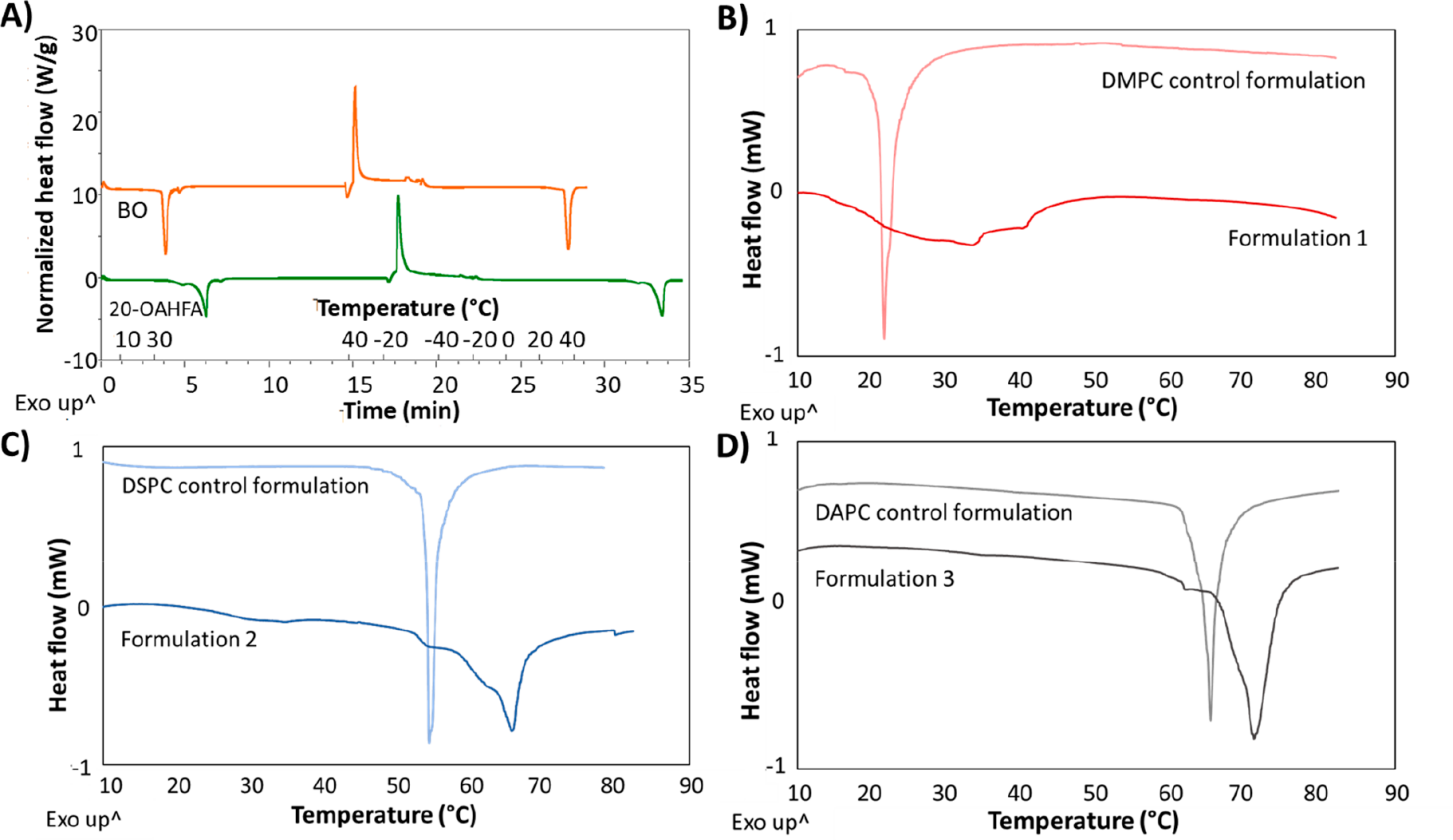
Phase transition behavior of BO (A), 20-OAHFA (A) and liposomal formulations (B–D) as characterized by DSC. For 20-OAHFA, two separate peaks were observed close to each other upon melting, and these were designated as *T*_m_1 (smaller peak) and *T*_m_2 (larger peak). Formulation 1: 4% DMPC/0.5% BO/0.5% 20-OAHFA; formulation **2**: 2% DSPC/0.5% BO/0.5% 20-OAHFA; formulation **3**: 2% DAPC/0.5% BO/0.5% 20-OAHFA. The control formulations are those in which the PC is formulated in the absence of 20-OAHFA and BO.

Although not mentioned earlier in the article, we did perform an in-depth thermal characterization of the formulations by differential scanning calorimetry (DSC) as part of the screening campaign. This formed the base for assessing when the 20-OAHFA and BO species were incorporated into the lipid bilayer (without the generation of separate subregions in the bilayer or free species remaining in the solution). In order to create a sound starting point for the assessment, we initially characterized the thermal behavior of the 20-OAHFA, BO, and individual PCs alone by DSC. A summary of melting temperatures for BO and 20-OAHFA (*T*_m_) and *T*_c_ values of liposomes are provided in Table 2, and representative thermograms are shown in Figure 1.

**Table 2:** Melting Temperatures (*T*_m_) of BO and 20-OAHFA and Gel-to-Liquid Phase Transition Temperatures (*T*_c_) of Liposomal Formulations^a^

active lipid	onset *T*_m_1 (°C)	peak *T*_m_1 (°C)	endset *T*_m_1 (°C)	onset *T*_m_2 (°C)	peak *T*_m_2 (°C)	endset *T*_m_2 (°C)
20-OAHFA^b^	42 ± 1	47 ± 0	48 ± 1	57 ± 1	60 ± 0	61 ± 0
BO	35 ± 0	37 ± 1	40 ± 0			

liposomal formulation	onset *T*_c_ (°C)	peak *T*_c_ (°C)	endset *T*_c_1 (°C)			
DMPC control formulation	17 ± 1	21 ± 0	28 ± 0			
DSPC control formulation	51 ± 1	54 ± 0	58 ± 2			
DAPC control formulation	64 ± 0	65 ± 0	69 ± 0			
formulation **1**	15 ± ND	ND	49 ± 2			
formulation **2**	53 ± 2	66 ± 0	68 ± 2			
formulation **3**	64 ± 5	71 ± 1	76 ± 1			

a Formulation **1**: 4% DMPC/0.5% BO/0.5% 20-OAHFA; formulation **2**: 2% DSPC/0.5% BO/0.5% 20-OAHFA; formulation **3**: 2% DAPC/0.5% BO/0.5% 20-OAHFA.

b For 20-OAHFA, two separate peaks were observed close to each other upon melting, and these were designated as *T*_m_1 (smaller peak) and *T*_m_2 (larger peak). ND: Not determined.

Both BO and 20-OAHFA induced clear exothermic peaks upon crystallization and endothermic peaks upon melting (Figure 1A). For 20-OAHFA, two separate peaks were observed close to each other upon melting, and these were designated as *T*_m_1 (smaller peak) and *T*_m_2 (larger peak). As for the formulations based on the PCs alone, the *T*_c_ values (endothermic peaks) were concordant with the ones reported by the manufacturer (Figure 1B–D). Incorporation of 20-OAHFA and BO in the phospholipid bilayer was accompanied by broadening of the gel-to-liquid phase transition peak (*T*_c_). In addition, this transition occurred at a higher temperature compared with the formulations without 20-OAHFA and BO. The thermograms were interpreted in the following way: if only a clear individual endothermic peak was detected by DSC, the active lipids were interpreted to be fully incorporated into the lipid bilayer as desired. In the case in which additional distinct endothermic peaks were present, these were interpreted as the inadequate incorporation of 20-OAHFA and BO (peaks overlapping with those of the pure compounds) or the possible generation of separate subregions in the lipid bilayer (peaks not overlapping with those of the pure compounds). It is worthwhile to note that this analysis was possible because the *T*_m_ and *T*_c_ values of BO, 20-OAHFA, and the formed liposomes do not significantly overlap.

With the DMPC-based formulations, we initially studied if there is a difference between incorporating either BO, 20-OAHFA or a combination of both on the thermal behavior of the formed liposomes. These studies were performed at several different ratios of BO, 20-OAHFA and DMPC. We did not discover any lipid specific changes in the thermal behavior of the liposomes (observation B, Table 1). However, the *T*_c_ area widened significantly as a function of the increased incorporation of BO and 20-OAHFA into the lipid bilayer (observation C, Table 1). The significant broadening of the *T*_c_ peak displayed by DMPC liposomes was considered to be an indication of a less unified phase transition due to higher heterogeneity in the lipid bilayer (Figure 1B). Due to the complex thermal behavior displayed by DMPC liposomes, we decided to assess the behavior of DSPC and DAPC liposomes before selecting representative formulations for further studies. The DSPC and DAPC based formulations displayed a similar phase transition behavior as noted above; however, with these PCs the broadening of the signal was more modest (Figure 1C and D). This allowed a more accurate assessment of successful incorporation of 20-OAHFA and BO species in the lipid bilayer. With both DSPC and DAPC based formulations, PC/BO/20-OAHFA mass ratios of 2:1:1 resulted in thermograms in which a small peak in close proximity to the *T*_m_ peak of BO could be detected (32–34 °C). Unsure of whether this peak corresponded to the incomplete incorporation of BO into the lipid bilayer (observation D, Table 1), or some other phenomenon such as the generation of a separate subregion in the lipid bilayer, we decided to limit further assessment to formulations in which the 20-OAHFA and BO were indicated to be fully incorporated in the lipid bilayer.

Altogether, the formulation development process enabled identification of the boundaries (total lipid concentration and the amount of 20-OAHFA and BO that can be incorporated in the lipid bilayer) within which these types of liposomal formulations can be successfully developed employing the thin film hydration protocol. Based on the formulation screening campaign, one representative formulation from each PC was chosen for in-depth biophysical and cellular assessment studies. The choice fell on relatively dilute liposomal formulations in which the amounts of BO and 20-OAHFA in relation to PC could be maximized. The three formulations will be referred to as formulation **1**: 4% DMPC/0.5% 20-OAHFA/0.5% BO, formulation **2**: 2% DSPC/0.5% 20-OAHFA/0.5% BO, and formulation **3**: 2% DAPC/0.5% 20-OAHFA/0.5% BO (Table 2 and Figure 1).

Additional factors of importance (pH, osmolality, and particle size) from the topical administration perspective were briefly considered at this point. These properties could be adjusted by modifying the liquid phase used during the formulation process. For example, if the lipid film was hydrated solely in water, the pH and osmolality of the formulations were not acceptable for topical administration to the eye (pH in the range of 3.4–4.3 and osmolality in the range of 4–28 mOsm/kg). However, by employing a tailored tris-buffered saline solution (50 mM TBS, pH 7.4) widely used in commercially available eye drops instead of water, formulations with neutral pH and close to isotonic osmolality (300–330 mOsm/kg) could be successfully produced. In addition, the average size of the liposomes were determined by nanoparticle tracking analysis (NTA) and were found to be in the acceptable 100–200 nm range in general (Supporting Table 1).

### 2.2 Characterization of the Biophysical Mechanism of Action

**Figure 2:**
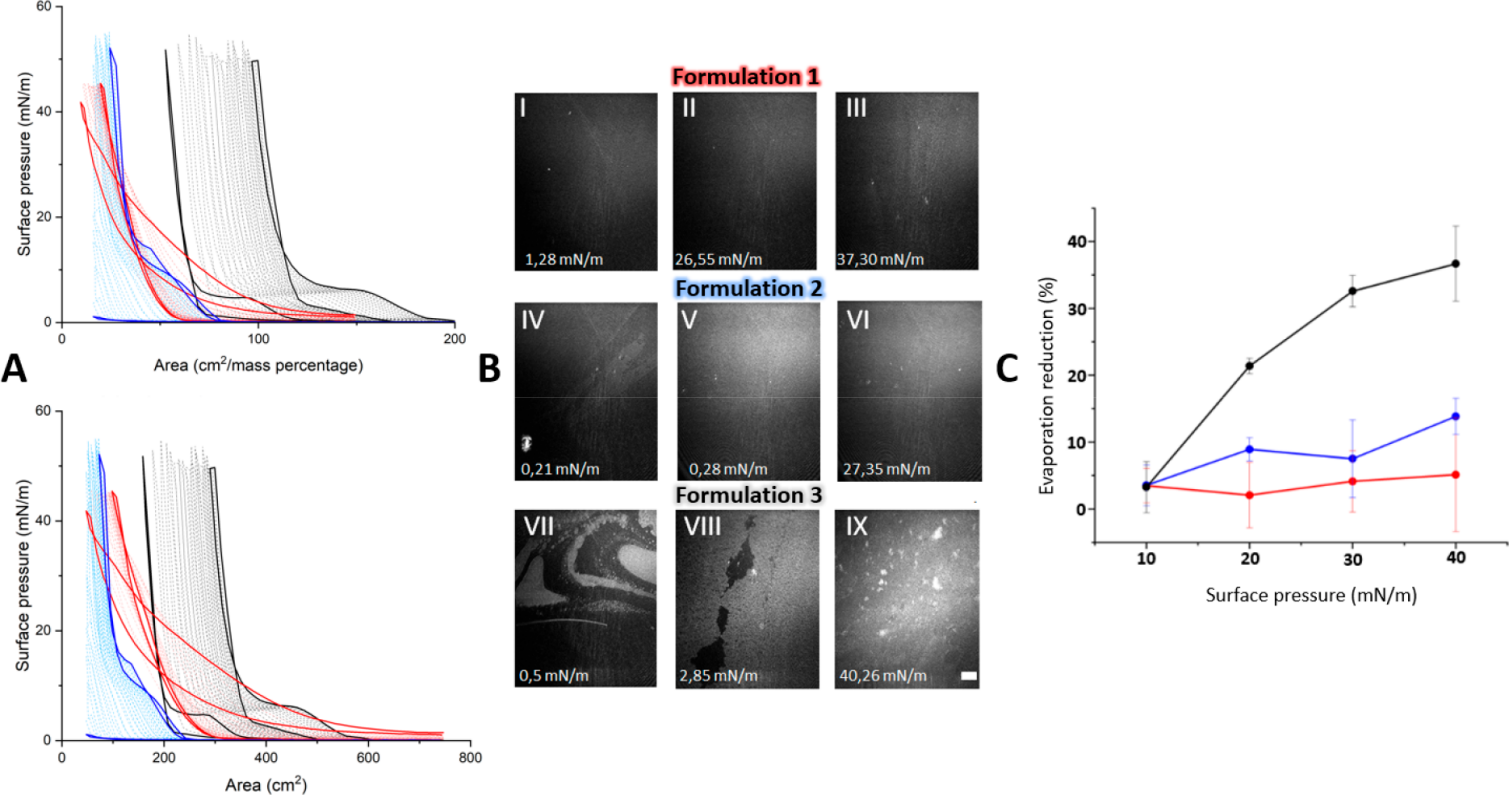
Excerpt from biophysical profiling studies. Formulation **1** is marked with red, formulation **2** with blue, and formulation **3** with gray/black in A–C. (A) Surface pressure isotherms over compression expansion cycles are showcased as a function of area (in either cm^2^ or cm^2^/mass percentage). The two bold lines represent the first (furthest to the left) and last (furthest to the right) measurements in the compression/expansion cycles. (B) BAM images highlighting the film structure are shown at representative surface pressures. The scale bar is 500 μm. (C) The evaporation reduction is presented as a function of the surface pressure.

In order to understand the behavior of the formulations when administered onto the ocular surface, we devised a protocol enabling the validation of key properties, such as spreading capability and evaporation reduction, under standardized experimental conditions. In more detail, we used a Langmuir trough setup in which the aqueous phase was tailored to mimic the electrolyte concentration and pH of the aqueous tear film layer (140 mM NaCl, 3 mM KCl, 10 mM phosphate buffer, pH 7.4, for more detail see the Experimental Section). The temperature was set to the physiological value of 35 °C and compression/expansion cycles coupled with Brewster angle microscopy (BAM) imaging could be utilized to monitor effects on lipid film structure/behavior during simulation of eye blink cycles. While this standardized Langmuir setup is uniquely suited to studying the film properties at the physiological ocular surface temperature and pressure range (20–40 mN/m), it cannot accurately recapture the blinking speed and frequency of the human eye. Nevertheless, the advantages of using a universal instrument which allows reproduction of findings from between distinct laboratories outweigh these limitations when it comes to the early stage biophysical profiling of formulations and individual lipid components. The results from our assessment of formulations **1**–**3** are summarized in Figure 2.

We started with the biophysical profiling of formulation **1**. Interestingly, the surface pressure lift off area was found to shift to larger areas during the compression–expansion cycles (Figure 2A). This shift was more pronounced during the early cycles and gradually transformed into a behavior with minor notable changes. Formulation **1** did not display a clear liquid to solid phase transition in the surface pressure isotherm and the BAM images did not provide evidence of the formation of a homogeneous solid phase although some solid speckles could be seen at high surface pressures (Figure 2B). Thus, the film remained for the major part in the liquid state throughout the compression/expansion cycles and especially in the ocular surface pressure range of 20–40 mN/m. Therefore, the liposome induced the formation of a lipid film on top of the aqueous phase with altered properties compared to those of films formed by BO and 20-OAHFA on their own. A control experiment was performed by dissolving DMPC, 20-OAHFA, and BO at identical ratios in chloroform and analyzing the surface behavior of the film formed by these constituents in their free form (Supporting Figure 1). The end outcome was similar (i.e. the properties of the lipid film formed at the aqueous interface were not significantly affected by the administration process (lipids in free form vs formulation **1**)). In order to understand the temperature dependence of these properties, the behavior of the lipid mixture in free form was assessed over the temperature range of room temperature to 40 °C (Supporting Information, Figure 2). We note that considerable changes in the biophysical profile were not uncovered through these studies thus suggesting that the lipid film retains its biophysical properties in this temperature range. On the whole, a sound indication that the mode of action of the lipid species are not affected by the source of their origin or the temperature. While we have in our earlier reports disclosed information on the correlation between film structure and evaporation-resistant properties and shown that films existing in the liquid state do not significantly reduce the evaporation rate of water from the underlying subphase,^24−26,34,35^ we proceeded by assessing the capabilities of formulation **1** (Figure 2C).

As expected, the film formed by formulation **1** did not have a statistically significant effect on the aqueous evaporation rate when compared to the control (aqueous layer without a lipid film covering it). While formulation **1** was capable of forming a lipid film at the aqueous subphase and spreading to cover an acceptable surface area, the film structure and related evaporation-resistant properties did not meet the expectations on an anti-evaporative dry eye product. Nevertheless, the respreading capabilities over compression/expansion cycles indicate that formulation **1** could potentially aid in spreading other lipid species at the aqueous interface. This could translate into successful targeting of the tear-film instability defect associated with DED by increasing the tear film break up time through improved coverage of the ocular surface by the tear film lipid layer. In this work, however, targeting the anti-evaporative defect in addition to the tear film instability defect was the goal, and we thus proceeded by evaluating formulations **2** and **3** under identical experimental conditions.

Both formulations **2** and **3** displayed a similar behavioral pattern across the compression/expansion cycles as formulation **1** (Figure 2A). While the surface pressure lift off area was found to shift to larger areas during the compression/expansion cycles, the lift off areas were found to vary between individual formulations. In addition, the gradual decrease in the shift observed for formulations **1** and **2** after the initial cycles was less pronounced for formulation **3**. These findings could indicate that the lipid film formed by formulation **3** continues to adapt over additional compression/expansion cycles compared to formulation **1** and **2** (i.e., over a prolonged time frame). While the DSC data suggested that the formulation would be stable under the employed experimental conditions, the fact that the surface pressure isotherms display a behavior reminiscent of that of BO/20-OAHFA mixtures suggest that they are released onto the aqueous surface to a certain extent. Additional studies focusing on the release kinetics and lipid trafficking between the subphase and surface will be required in the future to address these aspects in more detail. Nevertheless, we were pleased that the films formed by formulations **2** and **3** displayed a liquid to solid phase transition since a solid structure is required in order to enhance the evaporation resistance of the lipid film. Moreover, the behavior was similar for both formulations and the corresponding lipid compositions administered from chloroform solutions (Figure 2A and Supporting Figure 1). A temperature dependence on the properties displayed by these films could not be uncovered through studies of the mixtures spread from chloroform in the room temperature to 40 °C range (Supporting Figures 3 and 4). Nevertheless, the additional studies performed verified that the mode of action stems from the individual lipid species and that it is not negatively affected by the formulation process or significantly altered by temperature. While the surface pressure isotherms indicated that both formulations **2** and **3** were promising, the accompanying BAM images indicated that only the film formed by formulation **3** displayed the desirable solid structure in the physiological surface pressure range of 20–40 mN/m (Figure 2B). Next, the evaporation resistance of these films was assessed. The reduction in evaporation from the aqueous phase was found to be approximately 8% for formulation **2** and 30% for formulation **3** at 30 mN/m surface pressure (Figure 2C). The 30% reduction in evaporation of water from the aqueous layer is an excellent finding which proves that the targeted concept works.

Based on the screening program, a few factors emerged that warrant a more detailed discussion. First, the selection of PL proved crucial for obtaining a formulation that displays anti-evaporative properties. Here, tailoring the chain length of the PL-species was found to provide a suitable path toward optimizing these properties. However, all of the studied PC-species were found to have a detrimental effect on the evaporation reduction capabilities of the BO/20-OAHFA mixture. During the screening of evaporation-resistant properties displayed by PCs, BO and 20-OAHFA in free form, we could identify a negative trend in which an increase in PCs was accompanied by a decrease in evaporation resistance (Supporting Figure 5). This would insinuate that PLs are not the species responsible for the evaporation resistance of the natural intact TFLL (a topic under continuous debate), although further studies with a more diverse substrate scope will be needed to ascertain these factors. Our development program shows that careful selection of PL-species and lipid species can lead to liposomal formulations retaining promising respreading capabilities over compression/expansion cycles and anti-evaporative features. In other words, the liquid to solid phase transition occurring in formulation **3** leads to a formulation which promotes active spreading of lipids over compression/expansion cycles while simultaneously allowing tight packing of lipids in order to create an evaporation-resistant barrier. We consider that balancing these properties allows the development of optimal formulations for targeting two of the central functional defects caused by DED (tear film instability and increased evaporation of tear fluid defects). Understanding the “sweet spot” with respects to the structural features of lipids and the properties of their films will require building a bridge between the *in vitro* biophysical profiling performed here and future *in vivo* assessment studies. Simultaneously, a more diverse substrate scope must be assessed in order to strengthen the foundations of the assessment platform. Formulation **3** can serve as an invaluable standard going forward in this regard due to its unique and promising biophysical profile.

### 2.3 *In Vitro* Safety Studies in Human Corneal Epithelial Cells

**Figure 3:**
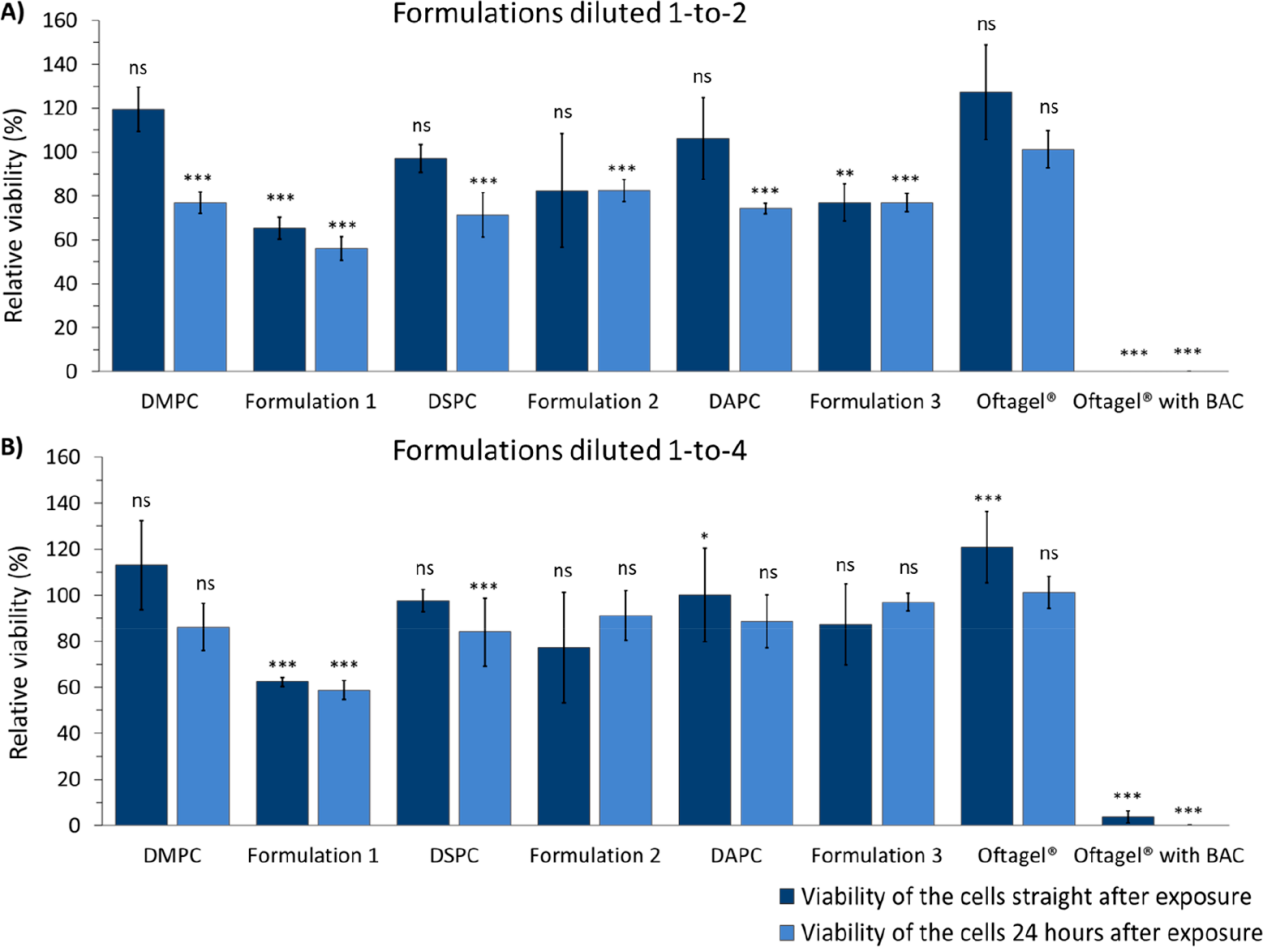
Relative viability % of HCE cells after 3 h exposure to the formulations. Formulations **1**–**3** were studied along with PC control formulations (DMPC, DSPC, and DAPC) and Oftagel (with and without BAC). (A) 1:2 dilution and (B) 1:4 dilution of formulations with serum-free medium. Control cells that were not exposed to formulations denoted the reference level of 100% cell viability. Viability was determined either straight after formulation exposure (dark blue bars), or after allowing them to recover for 24 h (light blue bars). The data is shown as mean ± standard deviation (*n* = 4–8). * *p* < 0.05, ** *p* < 0.01, *** *p* < 0.001, and ns: nonsignificant compared to nonexposed control cells.

The human corneal epithelial (HCE) cells were chosen for the safety assessment, as these represent the first ocular surface cells that the formulation would come in contact with. The effect of formulations on cell viability (i.e., safety of the formulations) was assessed by determining the dehydrogenase activity of the cells via a 3-(4,5-dimethyldiazol-2-yl)-2,5-diphenyltetrazolium bromide (MTT) assay after exposing the HCE cells to various concentrations of formulations for 3 h. The MTT assay was conducted either straight after formulation exposure and subsequent removal of formulations, or after allowing the cells to recover overnight (procedure described more thoroughly in section 4.5). Herein we exemplify the safety assessment by focusing on 1:2 and 1:4 dilutions. In the 1:2 dilutions (Figure 3), the greatest difference between formulations **1**–**3** was observed, while the 1:4 dilutions were representative for all other dilution ratios assessed (see Supporting Table 2 for more details).

We began by assessing the cytotoxicity of formulations **1**–**3**. Formulation **1** caused the most significant reduction in cell viability. The cell viability was 65%, or under, across the dilution range studied. For formulations **2** and **3**, the cell viability was found to be >77% across the dilution range studied and they can thus be considered as well tolerated on the cellular level. In order to ascertain these factors, we assessed the effect of the commercial ocular lubricant Oftagel in both its preservative free and preservative containing form in our assay (preservative: benzalkonium chloride (BAC)). The presence of BAC had a prominent effect on the results. In more detail, the preservative free Oftagel did not display negative effects on cell viability, whereas the BAC containing version had a dramatic effect on cell viability (essentially complete loss of cell viability) when dilution ratios of 1:2 and 1:4 were employed. At more diluted/dilute ratios, the preservative containing Oftagel was found to be better tolerated thus pointing toward a concentration-dependent cellular level toxicity. These results were expected since BAC is known to be toxic and imposes damage on epithelial cell membranes at high concentrations.^36−38^ Nevertheless, considering that both the preservative free and preservative containing versions of Oftagel are commercial dry eye products, formulations **1**–**3** (featuring PC, 20-OAHFA, and BO) as well as formulations featuring the PCs alone do not give rise to any safety related concerns on the cellular level.

### 2.4 *In Vitro* Efficacy Studies in HCE Cells

In addition to causing tear film instability and an increased evaporation rate of aqueous tear fluid (>80% of DED cases), DED is known to cause apoptosis of ocular surface epithelial cells.^9^ Therefore, we decided to investigate whether our formulations would be capable of promoting the recovery of HCE cells on top of the promising features identified through biophysical profiling studies. We set up an appropriate model to address these features. In more detail, the efficacy studies were performed by first engendering cell damage to the HCE cells with BAC, followed by treatment with the formulations in order to uncover potential beneficial effects. This experimental protocol was aligned with literature models as BAC is known to aggravate DED through various mechanisms^36,37^ and similar protocols are widely employed also in DED animal models.^38−40^ Thus, we envisioned that employing this *in vitro* model would give a sound foundation for assessing future crossover experiments *in vivo*.

**Figure 4:**
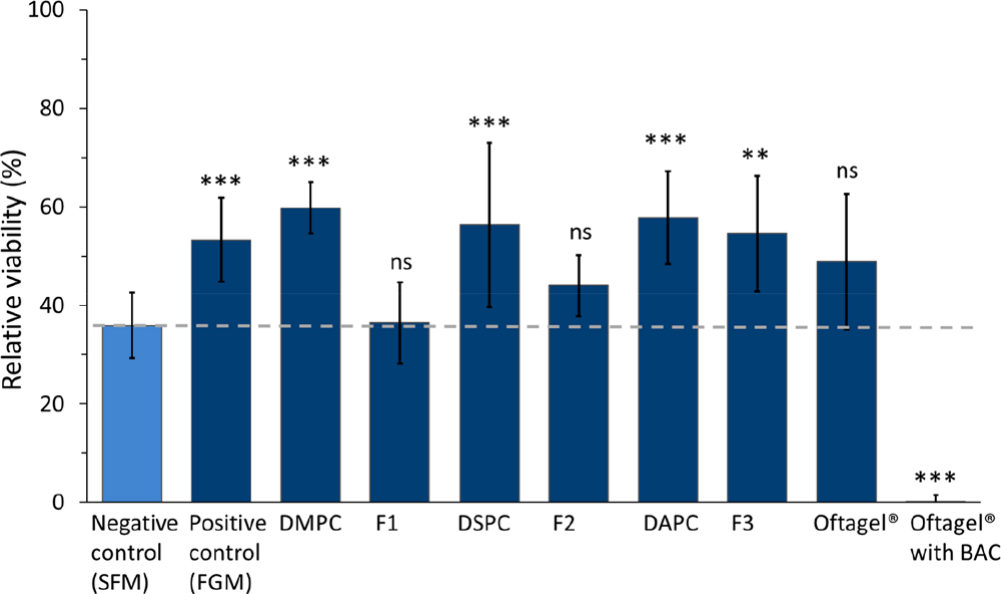
Recovery of the HCE cells after BAC induced cell damage when treated with the formulations for 24 h. Formulations **1**–**3** (F1–F3) were studied along with PC control formulations (DMPC, DSPC, DAPC) and Oftagel (with and without BAC). For the formulation treatment, all formulations were diluted 1:4 with serum-free medium (SFM). Control cells that were solely incubated in SFM (not exposed to BAC nor formulations) denoted the reference level of 100% cell viability, while the cells that were only exposed to BAC but not treated with formulations provided a baseline level (negative control) to which all other results were compared. The cells that were exposed to BAC and incubated in full growth medium (FGM) served a positive control for cell recovery. The data is shown as mean ± standard deviation (*n* = 6–9). * *p* < 0.05, ** *p* < 0.01, *** *p* < 0.001, and ns: nonsignificant compared to negative control (light blue bar).

The formulations were diluted in two ratios (1:4 and 1:8) with a serum-free medium (SFM). These ratios were chosen based on the cell viability assays (formulations deemed safe) and were considered to cover the most important range from the therapeutic perspective. We note that statistically relevant differences between the dilution series could not be uncovered and thus we focus on the results obtained with the 1:4 dilutions here (Figure 4). The results from the *in vitro* efficacy studies are summarized in Figure 4 and were interpreted in terms of observable general trends. Results from the other series are provided in Supporting Table 3. With formulations **1** and **2**, notable enhancements in cell recovery were not observed, even after a 24 h treatment period. However, for formulation **3** the trend indicated that it is capable of enhancing the cell recovery of HCE cells at a similar level as the positive control featuring cell growth medium (mean value indicates a 19% vs 17% increase in cell viability compared to the negative control). As expected based on the safety assay, a dramatic difference between the commercial Oftagel-products was witnessed. In more detail, the preservative free version functioned in a similar fashion as formulation **3** while the one containing BAC resulted in the complete loss of cell viability. The trend observed for the PC formulations without 20-OAHFA and BO indicated that the PCs may be the species responsible for promoting the recovery of HCE-cells (mean value indicates a 20–24% increase in cell viability compared to the negative control). In other words, in addition to allowing the successful development of a liposomal formulation containing 20-OAHFA and BO which displays good respreadability over compression/expansion cycles and evaporation-resistant properties, DAPC may contribute additional benefits to the final formulation. Intrigued by the early stage findings uncovered through the entire multidisciplinary *in vitro* screening platform reported herein, we are progressing to the final stages of the preclinical program which will focus on *in vivo* safety and efficacy studies.

## 3 Discussion

DED remains a considerable public health concern, which affects a large portion of the global population.^1^ There are currently no curing treatments on the market for DED and leading ophthalmologists have proclaimed that there is an immediate demand for new types of DED-treatments that successfully target the tear film instability and excessive tear evaporation defects caused by the disorder.^18^ In this work, we set out to develop a new treatment strategy that is capable of targeting the main defects caused by DED.

In more detail, inspired by the functional principle of the natural intact human TFLL, we used our previously tailored OAHFA and WE species as the base for the development campaign. Lipid films formed by 20-OAHFA and BO spread efficiently at the aqueous interface and packed tightly to form an outstanding evaporation-resistant barrier. We considered these properties to be essential for replenishing the mode of action of a dysfunctional TFLL by targeting the two central functional defects mentioned above.

Our formulation development campaign showed that the development of a liposomal formulation retaining these properties is possible; however, dedicated screening and careful selection of PLs is necessary in order to reach an appropriate balance between respreading capabilities over compression/expansion cycles and evaporation-resistant properties. This is because PL-films in general have been shown to display poor evaporation-resistant properties.^41^ Overall, we found that tailoring the chain lengths of endogenous PCs along with maximizing the incorporation of active lipid species into the phospholipid bilayer gave the best results. As the overall properties of the formulation are affected by all components and their proportional amounts, the lipid composition was optimized individually for each PC to achieve the best possible compromise of different features. With access to the most eligible formulations **1**–**3**, we setup a comprehensive early stage *in vitro* assessment platform which enabled the identification of the most promising candidate.

Characterization of the physical mode of action was accomplished by applying Langmuir monolayer techniques. While this setup is uniquely suited for verification of key properties such as spreading capacity and evaporation resistance, there are certain factors that this experimental setup is not able to recreate accurately. First, the slow movement speed of the barriers does not accurately capture the motion of an eye blink, which is considerably faster/more frequent and thus more efficient at spreading the formulations. Second, this setup is not able to recreate the natural environment of the ocular surface, including factors such as the flow of aqueous tears and the intrinsic dynamic interactions taking place in a complex and responsive living system. Nevertheless, the advantages of a standardized experimental setup allowing comparison and evaluation of key properties are central to the early stage development and identification of formulations with promising biophysical profiles.

Formulation **3**, featuring 20-OAHFA, BO, and DAPC displayed good respreading capabilities over compression/expansion cycles and reduced the evaporation of water by 30% at 30 mN/m. These are excellent findings, especially when taking into account that our approach is based on the functioning principle of endogenous TFLL lipids and therefore expected to have minimal disruptive effects on the other structural and functional features of the human tear film. Not only did formulation **3** demonstrate a remarkable biophysical profile, but also *in vitro* cytotoxicity and efficacy studies indicated that this formulation is safe to HCE cells and capable of promoting corneal epithelium recovery at the cellular level thereby successfully targeting other central defects caused by DED.

To conclude, through a comprehensive and multidisciplinary formulation development campaign we arrived at a promising formulation which may target the three main defects caused by DED: (1) stabilization of the tear film through active spreading of lipids, (2) reduction of water evaporation through tight packing of lipid species, and (3) promotion of damaged HCE cell recovery. Encouraged by these promising early stage findings, we are continuing on the development pipeline and will next focus on the *in vivo* safety and efficacy studies in animal models. The results of these studies will be reported in due course.

## 4 Experimental Section

### 4.1 Preparation and Characterization of the Active Lipids

BO is commercially available (purchased from Nu-Chek-Prep, Inc., MN), and 20-OAHFA was synthesized as previously described by our team.^24^ While the analytical data was in line with that reported earlier (HRMS, NMR, melting points, etc.), the NMR characterization was here carried out at a more detail level. The NMR spectra (^1^H, ^13^C, DQF-COSY, Ed-HSQC, HMBC) were recorded with a Bruker Avance III NMR spectrometer operating at 499.82 MHz (^1^H) and 125.68 MHz (^13^C). The probe temperature was kept at 25 °C. The spectra were processed in Bruker Topspin 4.0.7, and the chemical shifts and coupling constants in the ^1^H NMR spectra were further analyzed by quantum mechanical modeling using the Chemadder (Spin Discoveries Inc., Kuopio, Finland) software. In the reported data, the chemical shifts are expressed on the δ scale (in parts per million) using TMS (tetramethylsilane) or residual chloroform as internal standards. The coupling constants are given in Hz and provided only once when first encountered, whereas the coupling patterns are given as s (singlet), d (doublet), t (triplet), m (multiplet), etc. The more accurate NMR data for BO and 20-OAHFA are provided below. In addition, the purity of these two compounds was assessed by qNMR-techniques utilizing TraceCERT dimethyl sulfone (DMSO_2_) as an internal calibrant. The purity of both compounds exceeded 95%; see Supporting Information for details.

#### 4.1.1 Behenyl Oleate (BO)

^1^H NMR (499.82 MHz, CDCl_3_): δ 5.35 (dtt, 1H, *J*_9′,11′_ = −1.5, *J*_9′,8′_ = 7.2 Hz, *J*_9′,10′_ = 10.9 Hz, H-9′), 5.34 (dtt, 1H, *J*_10′,8′_ = −1.6, *J*_10′,11′_= 7.2 Hz, H-10′), 4.05 (t, 2H, *J*_1,2_= 6.7 Hz, H-1), 2.29 (t, 2H, *J*_2′,3′_ = 7.5 Hz, H-2′), 2.01 (ddt, 2H, *J*_8′,7′_ = 6.6 Hz, H-8′), 2.01 (ddt, 2H, *J*_11′,12′_ = 6.9 Hz, H-11′), 1.61 (tt, 2H, *J*_2,3_ = 7.9 Hz, H-2), 1.61 (tt, 2H, *J*_3′,4′_ = 6.5 Hz, H-3′), 1.38–1.19 (m, 58H, H-3–H-21, H-4′–H-7′, H-12′–H-17′), and 0.88 (each t, each 3H, each *J* = 6.7 Hz, H-22, H-18′) ppm. ^13^C NMR (125.68 MHz, CDCl_3_): δ 174.1 (C-1′), 130.1 (C-9′), 129.9 (C-10′), 64.6 (C-1), 34.6 (C-2′), 32.1 (C-16′, C-20), 29.9–29.2 (C-4–C-19, C-4′–C-7′, C-12′–C-15′), 28.8 (C-2), 27.4 and 27.3 (C-8′, C-11′), 26.1 (C-3), 25.2 (C-3′), 22.8 (C-17′, C-21), and 14.3 (C-18′, C-22) ppm.

#### 4.1.2 20-Oleoyloxy-eicosanoic Acid (20-OAHFA)

^1^H NMR (499.82 MHz, CDCl_3_): δ 10.83 (br s, 1H, COO*H*), 5.35 (dtt, 1H, *J*_9′,11′_ = −1.9, *J*_9′,8′_ = 7.3, *J*_9′,10′_ = 10.3 Hz, H-9′), 5.34 (dtt, 1H, *J*_10′,8′_ = −2.0, *J*_10′,11′_ = 7.2 Hz, H-10′), 4.05 (t, 2H, *J*_20,19_ = 6.7 Hz, H-20), 2.35 (t, 2H, *J*_2,3_ = 7.2 Hz, H-2), 2.29 (t, 2H, *J*_2′,3′_ = 7.6 Hz, H-2′), 2.01 (ddt, 2H, *J*_8′,7′_ = 6.9 Hz, H-8′), 2.01 (ddt, 2H, *J*_11′,12′_ = 6.7 Hz, H-11′), 1.63 (tt, 2H, *J*_3,4_ = 6.9 Hz, H-3), 1.62 (tt, 2H, *J*_19,18_ = 7.1 Hz, H-19), 1.61 (tt, 2H, *J*_3′,4′_ = 6.8 Hz, H-3′), 1.40–1.20 (m, 50H, H-4–H-17, H-4′–H-7′, H-12′–H-17′), and 0.88 (t, 3H, *J*_18′,17′_ = 6.7 Hz, H-18′) ppm. ^13^C NMR (125.68 MHz, CDCl_3_): δ 179.3 (C-1), 174.2 (C-1′), 130.1 (C-9′), 129.9 (C-10′), 64.6 (C-20), 34.6 (C-2′), 34.0 (C-2), 32.1 (C-16′), 29.9–29.2 (C-4–C-17, C-4′–C-7′, C-12′–C-15′), 28.8 (C-19), 27.4 and 27.3 (C-8′, C-11′), 26.1 (C-18), 25.2 (C-3′), 24.8 (C-3), 22.8 (C-17′), and 14.3 (C-18′) ppm.

### 4.2 Development of the Liposomal Formulations

Liposomal formulations were prepared utilizing three different PCs: DMPC, DSPC, and DAPC (purchased from Avanti Polar Lipids Inc., Alabaster, AL; purity stated by manufacturer > 99%). Overall, the goal was to incorporate a maximum amount of 20-OAHFA and BO into the liposomal phospholipid bilayer, without generating distinct separate subregions in the lipid bilayer, or compromising the stability and suitable flow properties from a dropwise application perspective.

Liposomal formulations were prepared by the thin film hydration method. PC, BO, and 20-OAHFA were dissolved in chloroform and mixed with various ratios, as illustrated in Table 1. The compositions were heated in a water bath above their phase transition temperatures (Table 2), and chloroform was evaporated (Rotavapor R-11; Büchi Labortechnik AG, Flawil, Switzerland) to create a thin lipid film on the inner surface of the flask. The lipid film was hydrated with 1–3 mL of water (all *in vitro* studies; sterile water or Milli-Q ultrapure Millipore, Bedford, MA) or 50 mM TBS (TRIS-buffered saline, pH 7.4, ThermoFisher Scientific) in the water bath. The sample was sonicated in an ultrasonic bath for 5–20 min (35 kHz, SONOREX SUPER RK 102 H, BANDELIN electronic GmbH & Co. KG, Berlin, Germany) or until the lipid film was visually detached from the surface of the flask. Further sonication with a probe sonicator for 2–5 min (20–25%/200 μm amplitude, SONICS Vibracell VCX750 Ultrasonic Processor with 1/2” probe and 2 mm tapered microtip, Sonics & Materials, Inc., Newtown, CT) was performed to reduce the liposome particle size. All steps from evaporating the chloroform to sonicating with the probe sonicator were done above the phase transition temperature of each formulation (Table 2).

### 4.3 Characterizations of the Liposomal Formulations

The liposomal formulations prepared were characterized by the different techniques highlighted below. Visual inspection was used to initially assess the potential of the prepared formulations. Formulations displaying undesired properties such as gel-like appearance were not further studied.

#### 4.3.1 Thermal Characterizations with Differential Scanning Calorimetry (DSC)

Differential scanning calorimetry (DSC 2500 with an RCS90 cooling unit; TA Instruments, Newcastle, DE) was utilized to study thermal properties of 20-OAHFA and BO, PC formulations from DMPC, DSPC and DAPC without 20-OAHFA and BO, and the PC formulations generated which included 20-OAHFA and BO.

The behavior of 20-OAHFA and BO was initially assessed. 1–4 mg of the individual lipids were weighted and placed into aluminum pans with a pierced lid (DSC Consumables incorporated, Austin, Minnesota). The pans were subsequently sealed and analyzed with DSC and TRIOS software (TA Instruments, Newcastle, DE). All results were compared to those of the similarly analyzed empty reference pan. Each measurement was performed in triplicate.

Briefly, the individual samples of 20-OAHFA and BO were equilibrated to 0 °C and then heated from 0 to 75 °C at a rate of 10 °C/min. The samples were kept isothermally at 75 °C for 10 min, after which they were equilibrated to −50 °C under an uncontrolled rate. Ultimately, the samples were heated again to 75 °C with a 10 °C/min heating rate. Nitrogen (N_2_) was utilized as the purge gas during these measurements (50 mL/min).

Next, the phase transition behavior of the PC liposomes in aqueous solution (with and without BO and 20-OAHFA) was studied by DSC to assess whether 20-OAHFA and BO were integrated into the phospholipid bilayer. The endothermic gel-to-liquid phase transition peaks for PC formulations without 20-OAHFA and BO were first determined and used as valid control. The concentrations of the control formulations detected with DSC were 4% (W/V) DMPC, 2% (W/V) DSPC, and 2% (W/V) DAPC. The generated liposomal formulations featuring 20-OAHFA and BO with suitable flow properties for dropwise application were then analyzed by DSC in a similar manner. In short, 20 μL of each formulation was pipetted into the aluminum pans. The concentrations employed were the same as those used in the formulation development process. For formulations **1**–**3**, these are given in supporting Table 1. The pans were subsequently sealed and analyzed with DSC employing the TRIOS software (TA Instruments, Newcastle, DE). The samples were cooled and equilibrated to 5 °C, after which they were heated from 5 °C to 70–85 °C at a rate of 10 °C/min. The range of 70–85 °C was set, because initial studies revealed that the lower temperature limit (70 °C) was not sufficient for all of the DSPC and DAPC based formulations (in order to obtain information on their phase transition behavior). The results were compared to those of the reference pan containing the liquid that was used to hydrate the lipid film. The thermograms were compared to those of the active lipids and control formulations. The absence of 20-OAHFA and BO peaks and phase transition behavior similar to those of the control formulations were interpreted as their successful integration into the phospholipid bilayer. The onset, peak, and endset temperatures were determined manually for all detected endothermic peaks.

#### 4.3.2 Nanoparticle Tracking Analysis (NTA)

The size of the liposome particles in the formulations were measured with ZetaView Nanoparticle Tracking Analyzer PMX-120-Z-520-F (Particle Metrix GmbH). All measurements were performed by using a 520 nm excitation laser at rt. For each size determination measurements, 11 cell positions were scanned and 30 frames captured with a camera sensitivity setting of 80 and shutter setting of 50 (i.e., duration of the exposure time of the camera 1/50 s). The suitable sensitivity for the measurements was selected by utilizing the software function ″Number of Particles vs Sensitivity″ to remove the background noise. In case abnormalities were detected in any of the 11 cell positions during the measurement, the individual cell position was removed from the final analysis. Formulation dilutions were made in a ratio that allowed the scattering intensity and the number of detected particles in one frame to be at an appropriate level (scattering intensity <8 and the number of detected particles 50–400). Accordingly, the samples were diluted 1:250 000–1:1000 000 with Milli-Q ultrapure water (Millipore, Bedford, MA) and injected into the measuring chamber. After the video capture, the videos were analyzed by the in-built ZetaView Software (version 8.05.14). The postacquisition parameters (i.e., the digital filters applied to images) for the analysis were set as follows: Maximum area 1000, minimum area 5, and minimum brightness 20.

#### 4.3.3 Assessing pH and Osmolality of the Formulations

The most eligible formulations were further characterized in terms of the pH and osmolality. The pH of the formulations was measured with an ORION SA 520 pH meter (Orion Research Incorporated, Boston, MA) which was autocalibrated with pH 4 and pH 7 buffer solutions (VWR chemicals, Radnor, Pennsylvania), while the osmolality of the formulations was measured with an Osmostat OM-6020 Auto-Osmometer (Daiichi Kagaku, Kyoto, Japan), which was calibrated with Milli-Q water and 300/1000 mOsm/kg standard solutions (Reagecon Diagnostics Ltd., Shannon, Ireland).

### 4.4 Evaluating Surface Activity and Evaporation Resistance of Mixtures and Formulations

The surface activity and the evaporation reduction of formulations and mixtures containing 5 mM 20-OAHFA, BO and DMPC, DSPC or DAPC in chloroform were studied with a KSV large Langmuir trough (Biolin Scientific, Espoo, Finland; dimensions 580 × 145 mm). A PBS-buffer (140 mM NaCl, 3 mM KCl, 10 mM phosphate buffer, pH 7.4) mimicking the aqueous tear film pH and electrolyte balance was used as a subphase. The PBS-buffer was prepared by the use of PBS-tablets (Medicago AB, Uppsala, Sweden) and Milli-Q water. The temperature was controlled with a circulating water bath (LAUDA ECO E4, Germany) and maintained at an ocular surface temperature of 35 ± 1 °C during the experiments. The surface pressure was determined with the Wilhelmy plate method, and BAM-imaging was performed with a KSV NIMA microBAM camera (Espoo, Finland).

The surface activity and spreading behavior of the formulations were assessed over 20 compression/expansion cycles. In short, 50 μL of the formulations was pipetted onto the subphase surface and the surface pressure was monitored with a Wilhelmy plate, while compression/expansion cycles (sinusoidal motion) were done with a barrier speed of 250 mm/min (92.5%/min from the initial area).

Changes in the film structure during the compression/expansion cycles were then studied in a separate measurement with the help of BAM-imaging. 150 μL of the formulations was pipetted onto the subphase, followed by compression/expansion cycles, during which the film was monitored with the equipment described above. During the cycles the barrier speed varied between 50–250 mm/min (18.5–92.5%/min from the initial area).

The ability to reduce the evaporation from the aqueous subphase was determined in the following way: 150 μL of the formulations was pipetted onto the subphase surface, whereafter compression/expansion cycles were performed with a barrier speed of 250 mm/min, until the formulations had spread to the subphase surface. The film was then compressed to a set surface pressure in the 10–40 mN/m (i.e., ocular surface pressure range) with a barrier speed of 50 mm/min(18.5%/min from the initial area), and a desiccant box filled with water absorbing silica gel was placed a few millimeters above the lipid film and kept in place for 5 min. A control measurement from the aqueous subphase as such was performed in order to obtain a valid reference point. After 5 min, the desiccant box was removed and weighted and the evaporation reduction caused by the formulation was calculated according to eq 1. Each measurement was performed four times, and the average value along with the standard deviations noting the experimental error margin are reported.
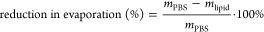
1In the equation: *m*_PBS_ is the mass absorbed by the desiccant in the absence of a formulation/lipid components while *m*_lipid_ is the mass absorbed by the desiccant in the presence of a formulation/lipid components.

### 4.5 *In Vitro* Cell Studies with Human Corneal Epithelial (HCE) Cells

#### 4.5.1 Culturing of HCE Cells

HCE cells were cultured at 37 °C and 5% CO_2_ using a growth medium consisting of 1:1 DMEM/F12 (Gibco by Life Technologies Limited, Paisley, UK), 15% FBS (Fetal bovine serum; Gibco by Life Technologies Limited, Paisley, UK), 100 U/mL penicillin, 0.1 mg/mL streptomycin (penicillin/streptomycin solution from EuroClone s.p.A, Pero, Italy), 0.3 mg/mL l-glutamine (EuroClone s.p.A, Pero, Italy), 10 ng/mL EGF (Recombinant Human Epidermal Growth Factor; Gibco by Life Technologies Corporation, Carlsbad, CA), 5 μg/mL human recombinant insulin (Gibco by Life Technologies Corporation, Grand Island, NY), 0.5% sterile filtrated DMSO (Sigma-Aldrich, St. Louis, MO), 0.1 μg/mL cholera toxin (Sigma-Aldrich, St. Louis, MO). Fresh growth medium was changed to the cells every 2–3 days. When reaching a 75–80% confluence, the cells were either subcultured 1:5–1:30 or seeded for the experiment. Upon confluence, the cells were washed with 1xDPBS (Gibco by Life Technologies Limited, Paisley, UK) and detached with 0.05% trypsin-EDTA (Gibco by Life Technologies Limited, Paisley, UK).

#### 4.5.2 *In Vitro* Safety Assessment by the 3-(4,5-Dimethylthiazol-2-y1)-2,5-diphenyl Tetrazolium Bromide (MTT) Assay

The *in vitro* toxicity of the most eligible formulations and control formulations was assessed in HCE cells using an MTT assay. This colorimetric method provides insights on the mitochondrial metabolic activity of the cells by correlating the detected absorbance to cell viability. The cultured HCE cells (passages 19 to 36) were seeded in 96-well plates (Costar 96 Well Cell Culture Plate, Corning Incorporated, Maine) at a density of 20 000 cells/well and after incubating overnight at 37 °C, the cells were exposed to serum-free medium (SFM) containing the formulations in various ratios (1:2–1:64 dilutions). Each formulation dilution was studied as a duplicate in two to four separate experiments (*n* = 4–8). In addition to the created formulations, also the effect of the commercial ocular lubricant Oftagel (Santen Pharmaceutical Co., Ltd.) were assessed in both its preservative free and preservative containing form. After incubating the cells with formulations at 37 °C for 3 h, the medium containing formulations was aspirated, and the cells were washed thoroughly with 1× DPBS to remove the remnants of the formulations. Thereafter, the cells were either allowed to recover overnight at 37 °C with only 150 μL of SFM or treated immediately with 100 μL of 0.5 g/L MTT medium. The MTT medium was prepared by mixing 10% of 5 mg/mL 3-(4,5-dimethylthiazolyl-2)-2,5-diphenyl tetrazolium bromide (MTT, Sigma-Aldrich, St. Louis, MO) in 1× PBS with 90% of SFM. 2 h after addition of the MTT medium, 100 μL of dodecyl sulfate sodium salt-*N*,*N*-dimethylformamide (SDS-DMF) lysis buffer (pH 4.7, 200 mg/mL SDS from Sigma-Aldrich, St. Louis, MO, in DMF/H_2_O 1:1, Gibco, ThermoFisher Scientific, Waltham, MA) was added to the wells with the following incubation overnight. Thereafter, the absorbance was measured at 570 nm by a Victor 2 multilabel plate reader (PerkinElmer, Wallac, St. Paul, MN). The percentage of cell viability was calculated as shown in eq 2. Wells that only consisted of MTT solution and SDS-DMF lysis buffer served as blanks and were subtracted from all samples. Wells with control cells that were not exposed to the formulations but merely cultured in SFM denoted the reference level of 100% cell viability.
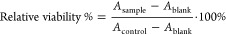
2where *A*_sample_ = absorbance of cells exposed to formulations, *A*_blank_ = absorbance of the reagent (without cells), and *A*_control_ = absorbance of cells exposed to serum-free medium (i.e., control).

#### 4.5.3 *In Vitro* Assessment of Formulation Efficacy with BAC Induced Cellular Model

The biological efficacy of the formulations was assessed with HCE cells by a benzalkonium chloride (BAC) induced model. In this experimental set up, the cells were initially exposed to BAC which is widely known to be toxic and induce corneal epithelial cell damage.^42−44^ With these studies, we wanted to assess whether our formulations enhance the HCE cell recovery after BAC induced damage as DED is also known to cause such defects. The cell viability was determined by the MTT assay, and the experimental setup was similar to the one described for the *in vitro* safety assessment described above. In short, after incubating the cells overnight at 37 °C in a 96-well plate, the cells were first exposed to 0.001% BAC for 45 min, and then after careful removal of BAC and thorough washing with 1× DPBS, the cells were treated with formulations diluted in SFM. Two different formulation dilutions (1:4 and 1:8) were used, both of which were studied in triplicate while the experiments were repeated in two to three separate times (*n* = 6–9). After incubating the cells at 37 °C for 24 h with formulations, the formulations were removed through thorough washing of the wells with 1× DPBS. Thereafter, they were subjected to the MTT assay. Again, the wells to which only the reagents had been added served as blanks, while control cells (cultured in SFM) that were not exposed to BAC nor formulations denoted the reference level of 100% cell viability. The cells only exposed to BAC but not further treated (but cultured in SFM) served as a negative control and baseline, while the cells that were treated with full growth medium (FGM) containing 15% FBS after BAC exposure served as a positive control, since FBS contains many growth promoting factors that enhance cell recovery.

#### 4.5.4 Statistical analysis

One-way analysis of variance (ANOVA) followed by Tukey’s multiple comparison test were used for assessing statistical significance of cell studies. The differences were considered as statistically significant when *p* < 0.05. These analyses were performed by GraphPad Prism 5.05 software (San Diego, CA).

## Supporting Information Available

Biophysical and *in vitro* data and a purity assessment of BO and 20-OAHFA (PDF)
