# Supplementary material for: Early-Stage Development of an Anti-Evaporative Liposomal Formulation for the Potential Treatment of Dry Eyes
Source: ACS Pharmacol Transl Sci. 2023 Sep 21;6(10):1518–30. doi: 10.1021/acsptsci.3c00147 (PMC10580384; doi:10.1021/acsptsci.3c00147)
Supplement: Supplementary file 1 — pt3c00147_si_001.pdf [file pt3c00147_si_001.pdf]

Supporting information

Early-stage Development of an Anti-Evaporative Liposomal Formulation for the Potential Treatment of Dry Eyes

Janika Jäntti,[℘,1] Tuomo Viitaja,[℘,2,3] Julia Sevón,[2] Tatu Lajunen,[1,4] Jan-Erik Raitanen,[2] Cordula Schlegel,[2] Mira Viljanen,[2] Riku O. Paananen,[2,3] Jukka Moilanen,[3] Marika Ruponen*[,1] and Filip. S. Ekholm*[,2]

[1] School of Pharmacy, University of Eastern Finland, P.O. Box 1627, FI-70211 Kuopio, Finland

[2] Department of Chemistry, University of Helsinki, P.O. Box 55, FI-00014 Helsinki, Finland

[3] Ophthalmology, University of Helsinki and Helsinki University Hospital, Haartmaninkatu 8, FI-00290 Helsinki, Finland

[4] Faculty of Pharmacy, University of Helsinki, FI-00790, Helsinki, Finland.

[℘]Equal contributions

* Corresponding author: marika.ruponen@uef.fi; filip.ekholm@helsinki.fi

Table of contents

1. The features of the formulations 1–3……………………….S2
2. Supporting biophysical data…………………………………….S2
3. Supporting cell viability data…………………………………..S5
4. Supporting efficacy data…………………………………………S6
5. Assessment of BO and 20-OAHFA purity by Q-NMR……..S6

1. The features of the formulations 1–3

Supporting Table 1. The lipid composition and size of the formulations **1**–**3**. Particle size (Diameter ± SD) determined with Nanoparticle tracking analysis (ZetaView® Nanoparticle Tracking Analyzer PMX-120 -Z-520-F, Particle Metrix GmbH, Inning am Ammersee, Germany).

Name	Lipid composition	% (W/V)	Molar ratios	Diameter ± SD (nm)
Formulation **1**	DMPC:BO:20-OAHFA	4:0.5:0.5	7:1:1	112.4 ± 48.4
Formulation **2**	DSPC:BO:20-OAHFA	2:0.5:0.5	3:1:1	152.9 ± 73.9
Formulation **3**	DAPC:BO:20-OAHFA	2:0.5:0.5	2.8:1:1	153.3 ± 65.2

2. Supporting biophysical data

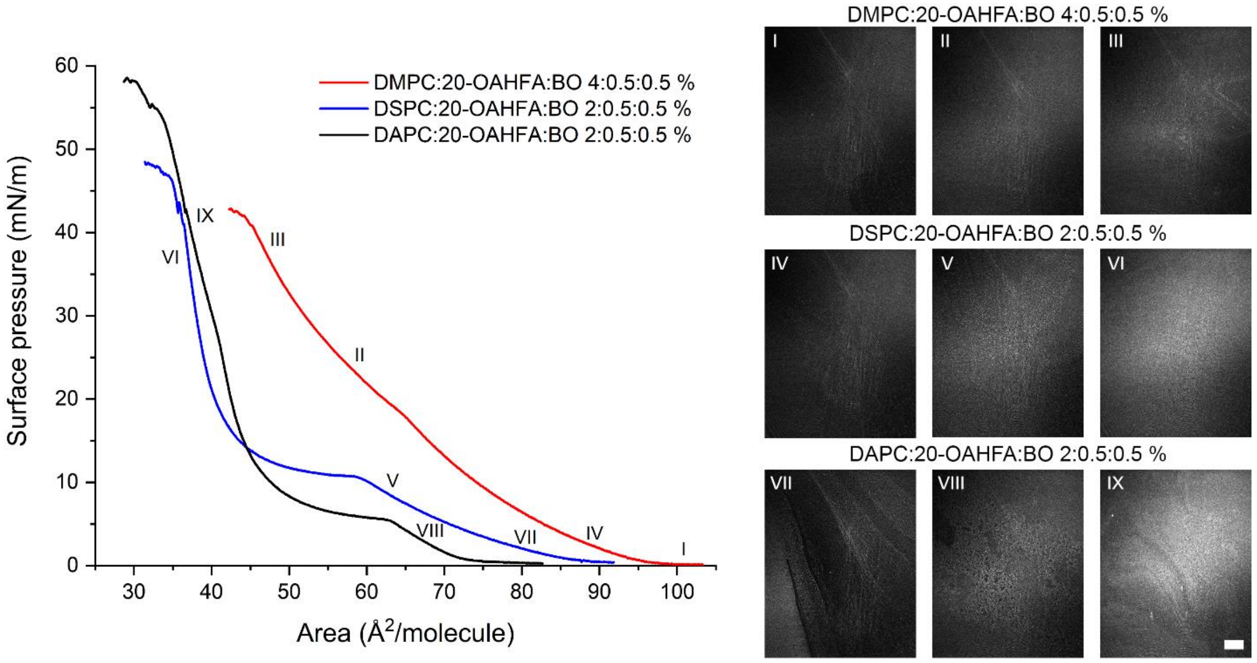

Supporting Figure 1. Studies on free lipids in chloroform solutions. **Left:** Surface pressure isotherms of corresponding PC:20-OAHFA:BO mixtures showcased as a function of area (Å^2/molecule). **Right:** Representative BAM images highlighting film structure at selected surface pressures. The scale bar depicts 500 μm.

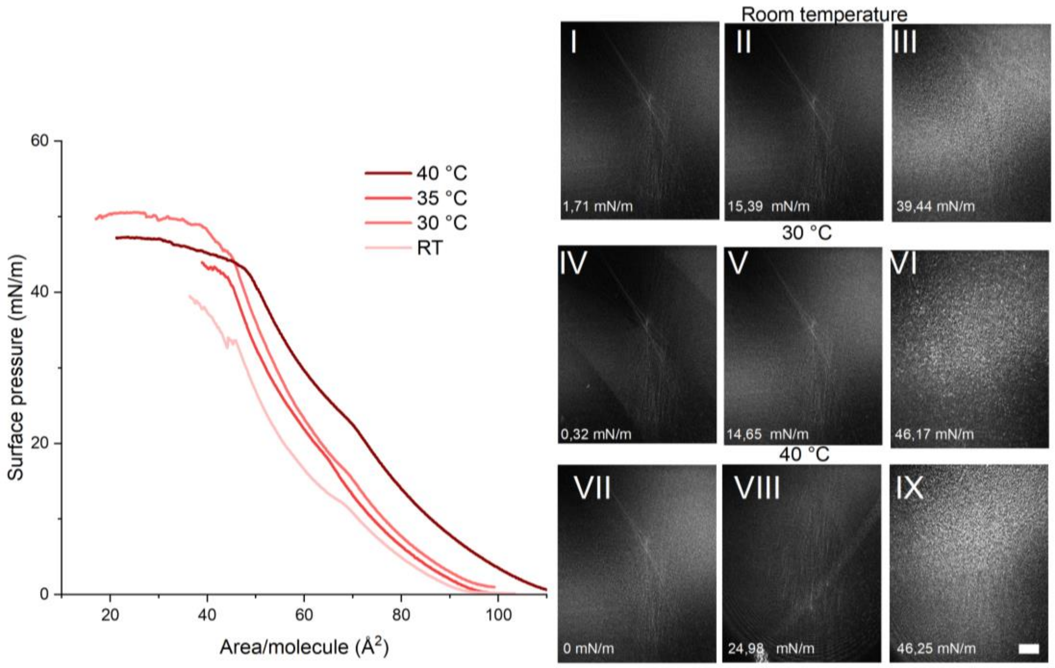

Supporting Figure 2. Temperature dependent behavior of DMPC:20-OAHFA:BO-mixture (4:0.5:0.5). **Left:** Surface pressure isotherms of DMPC:20-OAHFA:BO mixtures showcased as a function of area (Å^2/molecule). **Right:** Representative BAM images highlighting film structure at selected surface pressures. The scale bar depicts 500 μm.

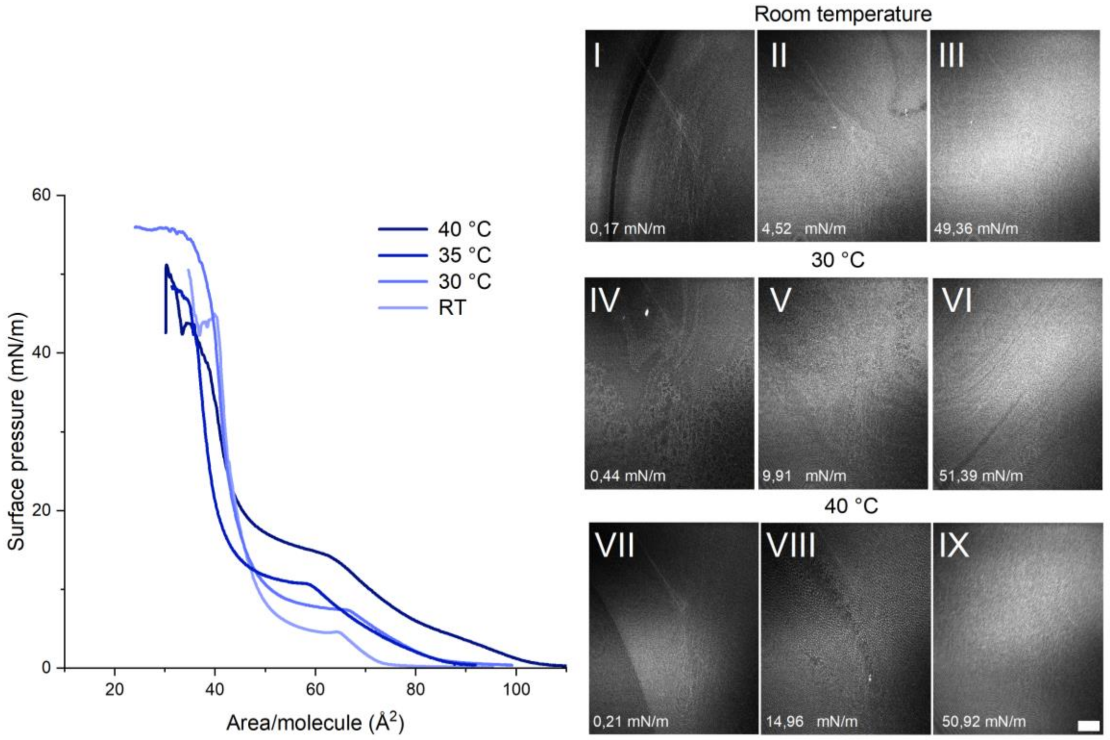

Supporting Figure 3. Temperature dependent behavior of DSPC:20-OAHFA:BO-mixture (2:0.5:0.5). **Left:** Surface pressure isotherms of DSPC:20-OAHFA:BO mixtures showcased as a function of area (Å^2/molecule). **Right:** Representative BAM images highlighting film structure at selected surface pressures. The scale bar depicts 500 μm.

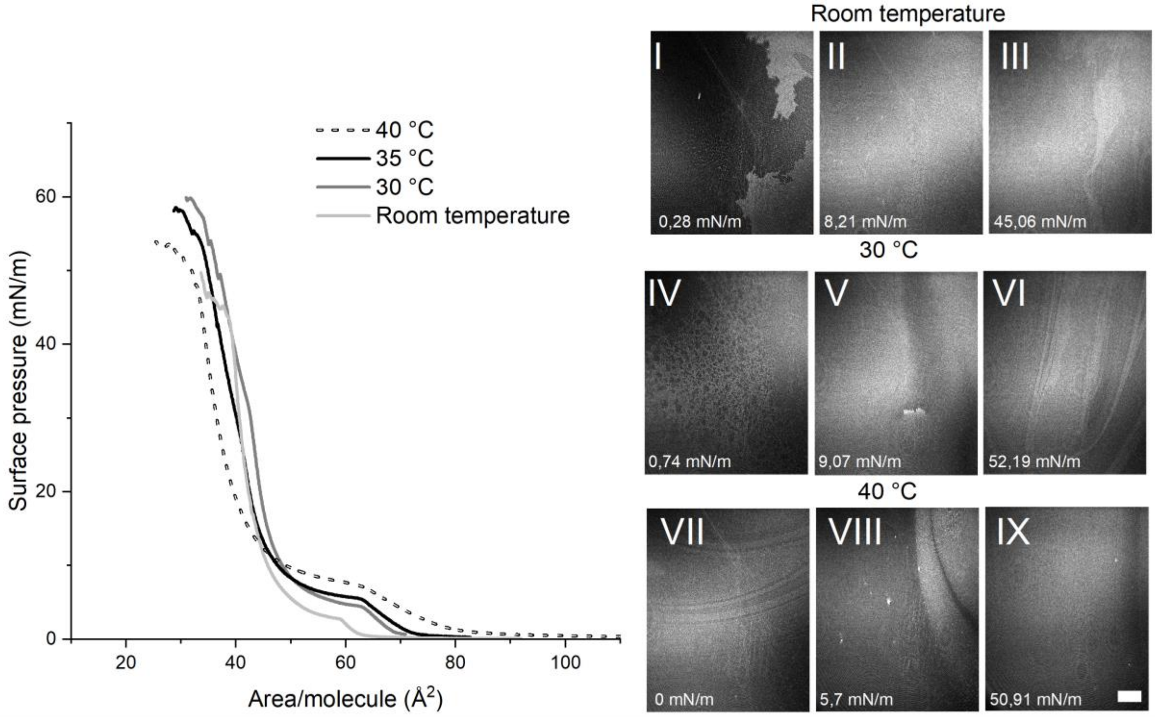

Supporting Figure 4. Temperature dependent behavior of DAPC:20-OAHFA:BO-mixture (2:0.5:0.5). **Left:** Surface pressure isotherms of DAPC:20-OAHFA:BO mixtures showcased as a function of area (Å^2/molecule). **Right:** Representative BAM images highlighting film structure at selected surface pressures.The scale bar depicts 500 µm.

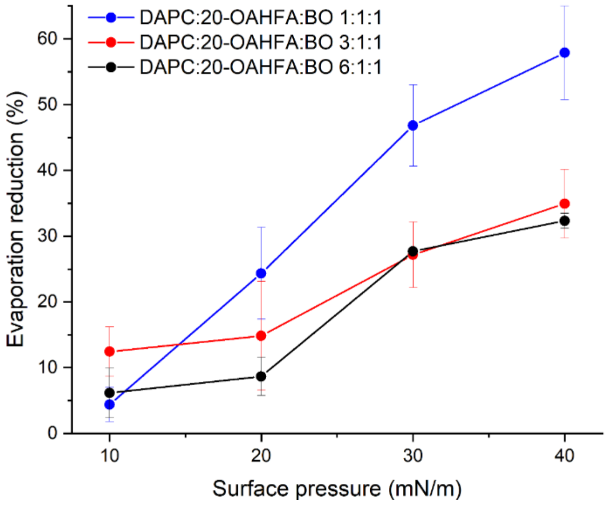

Supporting Figure 5. Assessment of the effects of representative DAPC:20-OAHFA:BO ratios on the evaporation reduction capacity.

3. Supporting cell viability data

Supporting Table 2. Relative cell viability % ± SD of human corneal epithelial cells after 3-hour exposures with various formulation dilutions. MTT assay conducted either straight after exposures or 24 hours after exposures (n = 4-8). Ns: No significance; * $p < 0.05$; ** $p < 0.01$; *** $p < 0.001$.

Formulation		**Relative viability ± SD (%)**					
		**1-to-2 dilution**	**1-to-4 dilution**	**1-to-8 dilution**	**1-to-16 dilution**	**1-to-32 dilution**	**1-to-64 dilution**
DMPC control formulation	Straight after exposure	119.5 ± 10.2 [ns]	113.0 ± 19.4 [ns]	122.0 ± 7.4 [ns]	114.0 ± 4.8 [ns]	112.5 ± 7.5 [ns]	113.2 ± 20.4 [ns]
	24 hours after exposure	69.2 ± 3.0 [***]	86.2 ± 10.2 [ns]	88.4 ± 7.2 [ns]	87.8 ± 7.1 [ns]	96.3 ± 10.0 [ns]	105.3 ± 2.0 [ns]
Formulation **1**	Straight after exposure	65.4 ± 5.0 [***]	62.3 ± 2.0 [***]	57.8 ± 3.1 [***]	51.8 ± 1.9 [***]	58.8 ± 3.4 [***]	64.2 ± 2.4 [***]
	24 hours after exposure	56.0 ± 5.4 [***]	58.8 ± 4.2 [***]	65.4 ± 7.1 [***]	61.8 ± 3.9 [***]	62.2 ± 0.4 [***]	64.6 ± 1.9 [***]
DSPC control formulation	Straight after exposure	97.1 ± 6.4 [ns]	97.7 ± 4.8 [ns]	97.9 ± 7.7 [ns]	98.3 ± 5.8 [ns]	97.6 ± 5.7 [ns]	91.5 ± 6.0 [ns]
	24 hours after exposure	71.4 ± 10.4 [***]	84.0 ± 14.7 [ns]	94.9 ± 11.9 [ns]	97.2 ± 15.5 [ns]	98.1 ± 23.1 [ns]	86.7 ± 18.1 [ns]
Formulation **2**	Straight after exposure	82.5 ± 26.0 [ns]	77.2 ± 23.9 [ns]	83.7 ± 22.1 [ns]	86.7 ± 21.4 [ns]	90.1 ± 17.1 [ns]	85.8 ± 18.3 [ns]
	24 hours after exposure	88.7 ± 12.3 [ns]	91.2 ± 10.7 [ns]	98.2 ± 8.9 [ns]	97.8 ± 7.0 [ns]	87.9 ± 7.9 [ns]	79.6 ± 12.2 [***]
DAPC control formulation	Straight after exposure	106.3 ± 18.6 [ns]	100.2 ± 20.4 [ns]	103.8 ± 15.9 [ns]	103.0 ± 20.1 [ns]	102.5 ± 24.1 [ns]	98.4 ± 20.3 [ns]
	24 hours after exposure	74.3 ± 2.5 [***]	88.6 ± 11.5 [*]	93.1 ± 5.6 [ns]	89.8 ± 6.6 [ns]	94.6 ± 7.4 [ns]	97.8 ± 5.7 [ns]
Formulation **3**	Straight after exposure	77.0 ± 8.5 [**]	87.4 ± 17.6 [ns]	92.3 ± 8.3 [ns]	83.4 ± 23.1 [ns]	84.6 ± 22.7 [ns]	80.8 ± 24.3 [*]
	24 hours after exposure	76.9 ± 4.8 [***]	97.0 ± 3.8 [ns]	94.4 ± 6.5 [ns]	95.6 ± 5.8 [ns]	103.2 ± 2.1 [ns]	96.6 ± 5.4 [ns]
Oftagel® single dose pipette	Straight after exposure	127.3 ± 21.6 [***]	120.9 ± 15.4 [***]	114.0 ± 15.4 [*]	111.8 ± 9.9 [ns]	95.2 ± 4.4 [ns]	Not studied
	24 hours after exposure	101.2 ± 8.5 [ns]	101.2 ± 6.9 [ns]	106.9 ± 11. 4[ns]	98.9 ± 10.0 [ns]	97.9 ± 4.4 [ns]	Not studied
Oftagel® bottle (Includes 0.06 mg/g BAC)	Straight after exposure	-1.6 ± 0.9 [***]	3.8 ± 2.6 [***]	75.5 ± 7.2 [***]	Not studied	Not studied	Not studied
	24 hours after exposure	-1.7 ± 1.8 [***]	-1.7 ± 0.9 [***]	32.5 ± 7.7 [***]	Not studied	Not studied	Not studied

4. Supporting efficacy data

Supporting Table 3. Relative cell viability % ± SD to illustrate the recovery of the HCE cells after BAC induced cell damage when treated with formulations for 24 h. Formulation treatments were conducted with two dilutions (1-to-4 and 1-to-8, n = 6-9). Ns: No significance; * p < 0.05; ** p < 0.01; *** p < 0.001 [compared to negative control], [[compared to dilution 1-to-4]].

**Sample**	**Relative viability ± SD (%)**	
Negative control	36.0 ± 6.7	
Positive control	53.3 ± 8.5 [***]	
	**1-to-4 dilution**	**1-to-8 dilution**
DMPC control formulation	59.8 ± 5.2 [***]	64.2 ± 3.5 [***] [[ns]]
Formulation **1**	36.5 ± 8.3 [ns]	37.7 ± 10.6 [ns] [[ns]]
DSPC control formulation	56.3 ± 16.7 [***]	54.2 ± 22.3 [**] [[ns]]
Formulation **2**	44.05 ± 6.2 [ns]	42.75 ± 12.8 [ns] [[ns]]
DAPC control formulation	57.8 ± 9.4 [***]	57.0 ± 10.9 [***] [[ns]]
Formulation **3**	54.6 ± 11.7 [**]	52.7 ± 10.5 [*] [[ns]]
Oftagel® without BAC	48.9 ± 13.8 [ns]	56.1 ± 16.5 [***] [[ns]]
Oftagel® with 0.06 mg/g BAC	0.1 ± 1.4 [***]	- 0.7 ± 0.6 [***] [[ns]]

5. Assessment of BO and 20-OAHFA purity by qNMR

Sample preparation: BO or 20-OAHFA and *Trace*CERT® dimethyl sulfone ($DMSO_2$; standard for quantitative NMR, CAS#: 67-71-0) were weighed into a 5 mm NMR-tube and dissolved in 0.7 ml of deuterated chloroform (Eurisotop) containing 0.03 % of TMS. Directly after preparation of the NMR-sample, the tubes were capped and NMR recorded.

NMR instrumentation and parameters: The ^{1}H-NMR spectra used in the determination of compound purity were recorded with a Bruker Avance III spectrometer (operating at 499.82 MHz (^{1}H)) equipped with a 5 mm BBFO^{plus} *Z*-gradient high resolution probe. The zg pulse program was employed. The probe temperature was kept at 25 °C. Matching, tuning, shimming and pulse calibration was performed by the topspin commands atmm, topshim and pulsecal. Other relevant parameters: 132 k data points, 262 k zero-filling (SI), 2 dummy scans, 16 scans. Processing of spectra was performed with Bruker Topspin 4.0.7. Fourier transformation of the spectra were performed using a line broadening of 0.3 Hz. Automatic phase correction and baseline correction was performed by the commands apk and abs. The spectra were calibrated according to the residual solvent peak or TMS. Information regarding the signals of interest used for the quantification and their integrals is provided below while calculating the purity of each of the two lipid species.

Calculation of sample purity: Adequately high S/N-ratios (>10) were observed in both spectra. First, the compound spectra were completely characterized and each signal assigned by a combination of 1D (^{1}H, ^{13}C) and 2D-NMR spectroscopic techniques (COSY, Ed-HSQC and HMBC) and the coupling constants of all signals were determined by the use of the ChemAdder software which applies quantum mechanical optimization in the spectral simulations. This information is available in the

experimental section. We then proceeded by assessing the purity through the protocol outlined by Pauli, G. F. et al. in the manuscript entitled "Importance of Purity Evaluation and the Potential of Quantitative 1H NMR as a Purity Assay" and published in *J. Med. Chem.* **2014**, *57*, 9220–9231. As mentioned above, we used *Trace*CERT® $DMSO_2$ as the internal calibrant because it has a good solubility in $CDCl_3$ and it gives rise to a singlet in the ^{1}H-NMR spectrum which does not overlap with those of the lipid species. According to the qNMR protocol employed, the purity of both starting materials exceeded 95%. The detailed calculations and the integrated spectra are provided below.

Behenyl oleate (BO):

The CH_3-signals in both BO and $DMSO_2$ were utilized in the qNMR-assessment.

Step 1:[a] m_s = 8.4 mg, m_{IC} = 2.0 mg, P_{IC} = 0.99 %

[a] Both compounds were weighed directly in the NMR-tube

Step 2: Int_t = 5.9518 (6 H, 0.95–0.82 ppm), n_t = 6

Step 3: Int_{IC} = 8.7174 (s, 6 H, 3.14–2.81 ppm), n_{IC} = 6

Step 4: MW_t = 590.60 g/mol, MW_{IC} = 94.13 g/mol

Step 5: P [%] = $(n_{IC}\times Int_t\times MW_t\times m_{IC})\times P_{IC}$ / $(n_t\times Int_{IC}\times MW_{IC}\times m_s)$

P [%] = (6×5.9518×590.6 g/mol×2.00 mg)×0.99 / (6×8.7174×94.13 g/mol×8.4 mg) = **101 %**

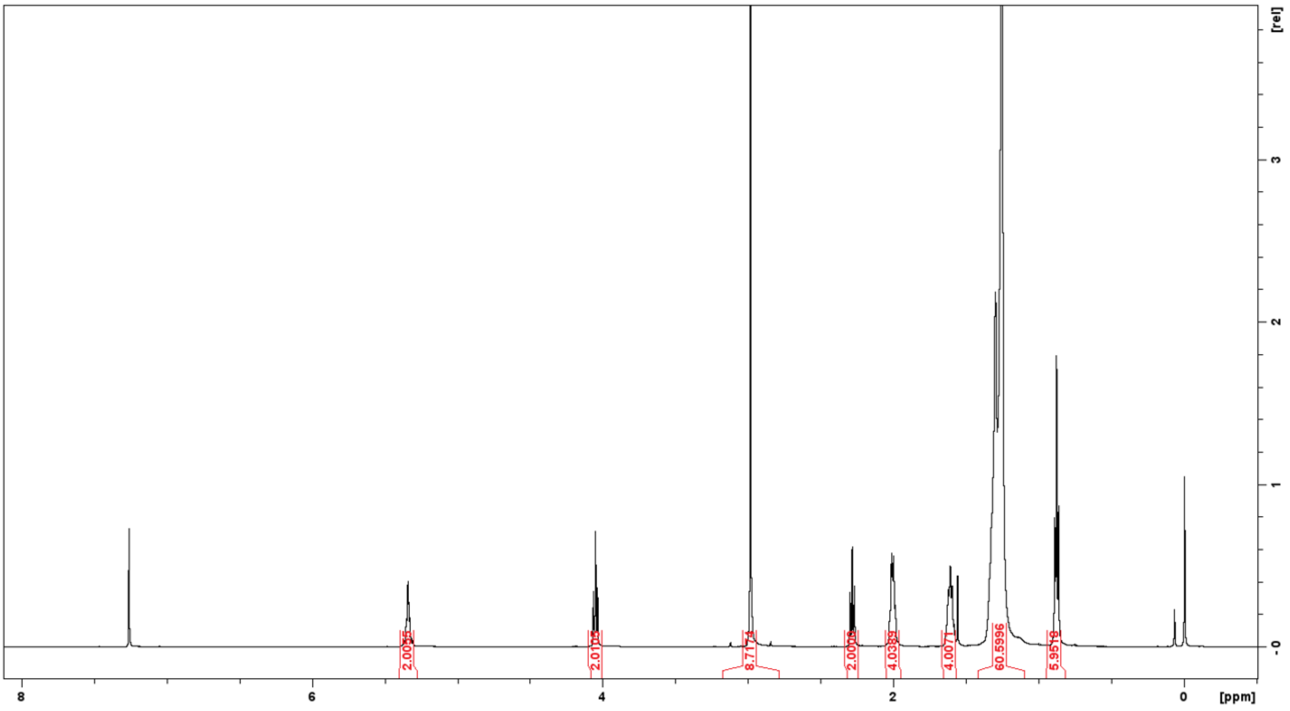

Supporting Figure 6. The integration values used for absolute qNMR of BO.

20-Oleoyloxy-eicosanoic acid (20-OAHFA)

The CH_3-signals in both BO and $DMSO_2$ were utilized in the qNMR-assessment.

Step 1:[a] m_s = 3.1 mg, m_{IC} = 4.1 mg, P_{IC} = 0.99 %

[a] Both compounds were weighed directly in the NMR-tube

Step 2: Int_t = 2.9466 (3 H, 0.91–0.85 ppm) , n_t = 3

Step 3: Int_{IC} = 48.26 (6 H, 3.14–2.81 ppm), n_{IC} = 6

Step 4: MW_t = 592.54 g/mol, MW_{IC} = 94.13 g/mol

Step 5: P [%] = $(n_{IC} \times Int_t \times MW_t \times m_{IC}) \times P_{IC} / (n_t \times Int_{IC} \times MW_{IC} \times m_s)$

P [%] = (6×2.9466×592.54 g/mol×4.1 mg)×0.99 / (3×48.26×94.31 g/mol×3.1 mg) = **100 %**

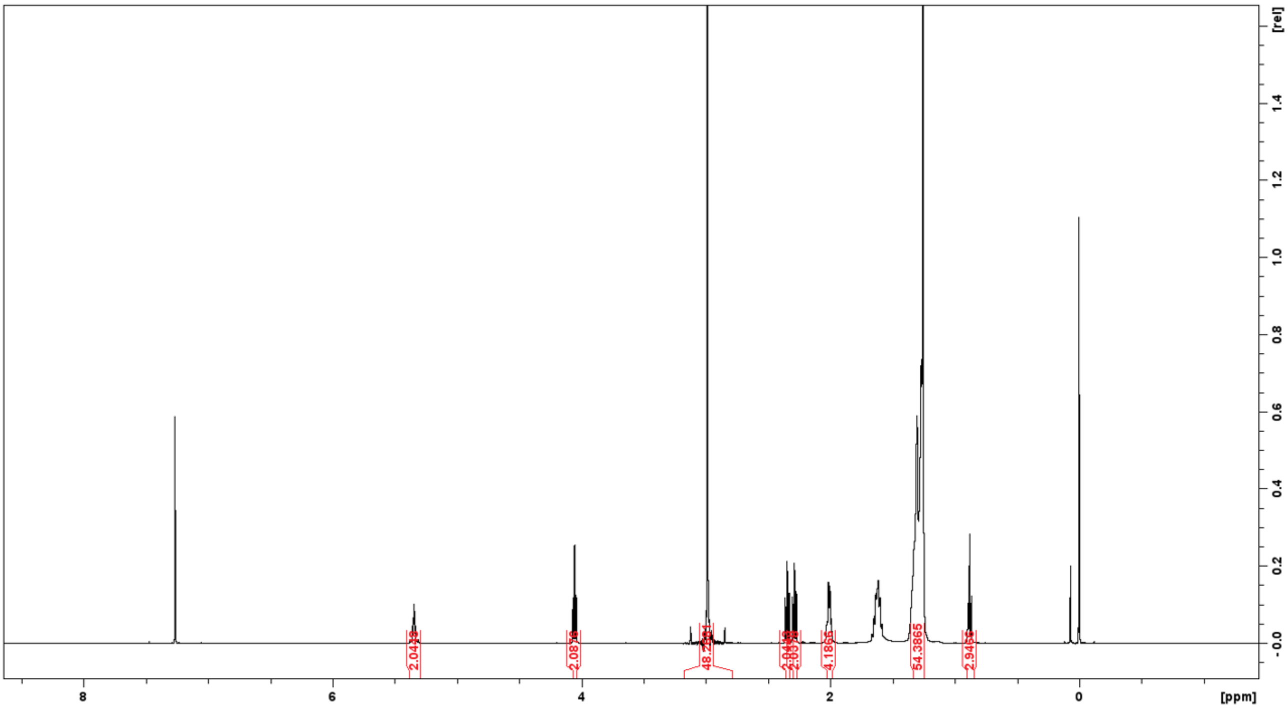

Supporting Figure 7. The integration values used for absolute qNMR of 20-OAHFA.
